# Association of valproate use and hippocampal atrophy in idiopathic generalized epilepsy

**DOI:** 10.1016/j.nicl.2025.103744

**Published:** 2025-01-27

**Authors:** Xiang Huang, Yingying Zhang, Qiuxing Lin, Kailing Huang, Yuming Li, Peiwen Liu, Danyang Cao, Wenhao Li, Wei Li, Xiuli Li, Qiyong Gong, Dong Zhou, Dongmei An

**Affiliations:** a Department of Neurology, West China Hospital, Sichuan University, Chengdu, Sichuan, China; b Department of Geriatric Medicine, West China Hospital, Sichuan University, Chengdu, Sichuan, China; c Huaxi MR Research Center (HMRRC), Department of Radiology, West China Hospital of Sichuan University, China

**Keywords:** Valproate, Hippocampus, Idiopathic Generalized Epilepsy, HippUnfold

## Abstract

**Objective:**

Recent studies revealed the effect of valproate (VPA) on brain structural changes in idiopathic generalized epilepsy (IGE). We aimed to investigate the volume of the entire hippocampus and subfields in patients with IGE, and explored their associations with VPA use.

**Methods:**

A total of 211 patients with IGE and 97 healthy controls (HCs) were enrolled in this study. All participants underwent T1-weighted images. Each hippocampus was segmented into seven subfields using HippUnfold. The volumes of bilateral hippocampi and each hippocampal subfield were evaluated. Spearman correlation analyses were performed to identify VPA use related abnormalities in IGE. Subgroup analyses for juvenile myoclonic epilepsy (JME), epilepsy with generalized tonic–clonic seizures alone (GTCA), and absence epilepsy (AE) were conducted.

**Results:**

The volumes of bilateral hippocampi were reduced in IGE compared with HCs. Subgroup analysis showed significant volume reductions in right hippocampus and its subfields in GTCA. Additionally, significant volume reductions were detected in bilateral hippocampal volumes and subfields in IGE patients currently taking VPA compared with HCs. A negative correlation was observed between the left CA2 volume and the age of onset.

**Conclusions:**

Our study revealed volume reductions in bilateral hippocampi in IGE, as well as in the right hippocampus and its subfields in GTCA. Abnormalities in both subfields and the whole hippocampus were associated with VPA use. These findings suggest that VPA may have more extensive neuroanatomical effects in IGE, potentially accounting for the heterogeneity observed in neuroimaging studies.

## Highlights

• HippUnfold was applied to segment hippocampus.
• The volumes of bilateral hippocampi were reduced in idiopathic generalized epilepsy compared with healthy controls.
• Subgroup analysis showed significant volume reductions in right hippocampus and its subfields in generalized tonic–clonic seizures alone.
• Valproate use was associated with hippocampal atrophy in IGE patients.

## 1 Introduction

Idiopathic generalized epilepsy (IGE) represents a prevalent form of epilepsy characterized by recurrent seizures of generalized onset and constitutes nearly one-fifth of all patients attending epilepsy clinics (Devinsky et al., 2024, Jallon and Latour, 2005). IGE is a relatively heterogeneous syndrome, including four subgroups: namely childhood absence epilepsy (CAE), juvenile absence epilepsy (JAE), juvenile myoclonic epilepsy (JME), and epilepsy with generalized tonic–clonic seizures alone (GTCA) classified by the International League Against Epilepsy (ILAE) (Scheffer et al., Apr 2017). Previous neuroimaging studies have reported brain structural changes in IGE, including the thalamus, hippocampus, frontal lobe, etc., but with considerable variations (Bin et al., 2017), which was often explained by the differences in study design, imaging methodology, sample sizes, as well as heterogeneities in demographic and clinical characteristics across studies. However, limited studies have emphasized the influence of antiseizure medications (ASMs) on brain structural morphology.

Valproate (VPA) is one of the most prescribed ASMs in IGE. It was noted that prior neuroimaging investigations have highlighted cortical thinning in the frontal lobe and the posterior quadrant associated with current VPA utilization in patients with epilepsy (Pardoe et al., 2013, Tondelli et al., 2020, Crespo Pimentel et al., 2024). A recent voxel-based morphometry (VBM) study (Shin et al., Jan 2024) detected widespread gray matter (GM) volume reduction in bilateral cerebellum and hippocampus, as well as in other brain regions in patients with IGE receiving VPA treatment, underscoring the need to consider VPA use as a potential confounding factor in morphometric MRI studies. A longitudinal study has also revealed accelerated brain volume loss over 1 year in both the hippocampus and the whole brain in patients with Alzheimer's disease treated with VPA compared with those not (Fleisher et al., 2011). Thus, we hypothesize that brain volume alteration, particularly hippocampal atrophy may be associated with VPA administration in IGE. However, the presence of hippocampal atrophy remains controversial in IGE. Some studies have reported significant left (Caciagli et al., 2019) or right (Lin et al., Oct 2013) hippocampal volume reduction or no alteration (Knake et al., Nov 2017) in patients with JME compared with healthy controls (HCs). Additional comparative studies focusing on GTCA have underscored more pronounced volume reductions in left or bilateral hippocampi relative to HCs (Zhou et al., 2015, Bochyńska et al., 2022). In addition, no related studies have been conducted so far to explore the relationship between VPA use and microstructural alterations of the hippocampus in IGE.

Therefore, our study aimed to investigate the association of VPA use and microstructural alterations in the entire hippocampus and its subfields among a considerable sample of patients with IGE and a group of HCs. One of the advanced neuroimaging techniques, namely HippUnfold, was a segmentation tool applied in the current study, which not only provided subfield volumes and morphological details, but also enabled measurements of the thickness of the entire anterior-posterior axis of the hippocampus, providing enhanced anatomical details and greater precision through topological alignment across individuals with varying anatomical structures and shedding light on potential alterations associated with epilepsy (DeKraker et al., 2022). Additionally, the relationship between hippocampal volume alterations and clinical characteristics was also examined in patients with IGE.

## 2 Material and Methods

### 2.1 Study population

A total of 211 right-handed patients with IGE were recruited from the Epilepsy Center at West China Hospital, Sichuan University. The diagnosis of specific IGE syndrome was based on the current ILAE criteria (Hirsch et al., Jun 2022). Exclusion criteria were the following: (1) a history of psychiatric disorders (such as schizophrenia, interictal psychosis, bipolar disorder, etc.) or any other neurological conditions; (2) substance addiction history; (3) visible abnormities in structural MRI; (4) any contraindications to MRI scanning. Detailed individual demographic information, clinical and routine MRI scan and scalp electroencephalography results were collected. Demographic and clinical evaluations included age at seizure onset, duration of epilepsy, comprehensive medical history and seizure semiology. Additionally, ninety-seven right-handed HCs, matched for age and gender, were recruited, all of whom had no abnormal MRI findings or history of neurological or psychiatric disorders. The Hamilton Anxiety Rating Scale (HAMA) (Maier et al., 1988) and Hamilton Depression Rating Scale (HAMD) (Ballesteros et al., Sep 2007) were performed to obtain for anxiety and depression scores; respectively.

### 2.2 MRI data acquisition

All participants underwent MRI scanning by a 3T scanner (Trio, Siemens) with an eight-channel head coil. T1-weighted images were obtained using 3D magnetization prepared rapid acquisition gradient echo (MPRAGE) sequence with the following parameters: repetition time (TR): 2300 ms; echo time (TE): 4.18 ms; flip angle: 9°; field of view (FOV): 256 × 256 mm^2^; voxel size: 1.0 × 1.0 × 1.0 mm^3^. Foam padding and earplugs were used to reduce head motion and scanner noise.

### 2.3 Volumetric analysis of hippocampus and total intracranial volume (TIV) acquisition

**Fig. 1:**
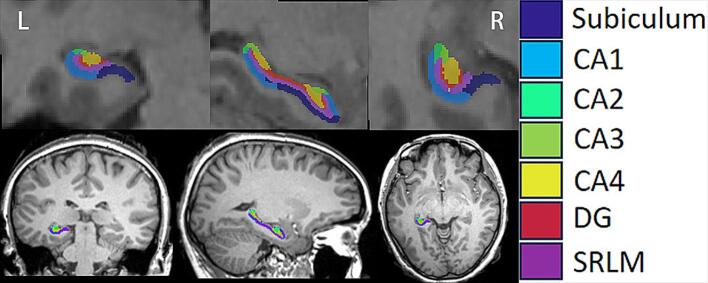
An example of seven subfields of left hippocampus segmented by HippUnfold in one patient with IGE. Abbreviations: CA, Cornu Ammonis; DG, dentate gyrus; SRLM,stratum radiatum, Lacunosum, and moleculare.

The newly automated segmentation software, HippUnfold (Version 1.4.1, https://hippunfold.readthedocs.io/en/latest/), was used in this study for surface-based segmentation and unfolding of the hippocampus (DeKraker et al., 2022). By using T1-weighted MR images, each side of the hippocampus was segmented into seven subfields (Fig. 1): Subiculum (Sub), Cornu Ammonis (CA)1–4, Dentate Gyrus (DG); Stratum radiatum, Lacunosum, and moleculare (SRLM). HippUnfold provided volume outputs for each subfield in mm (Scheffer et al., Apr 2017) of bilateral hippocampi. All subfield segmentations for both hemispheres were reviewed for gross errors by XH and YYZ，and any segmentation results judged to be incorrect were excluded. To test whether volume changes were associated current VPA use and disease duration in patients with IGE. Patients were first categorized into two groups based on their current ASMs: those who were currently taking VPA (VPA+ group) and those who were not currently taking VPA (VPA– group). The VPA– group was further divided into past VPA users and patients who had never used VPA (never VPA users). Based on the median dosage of VPA taken in the current patient cohort, we divided patients into two groups: IGE VPA1 and IGE VPA2. Then, we compared the volumes of bilateral hippocampi and each hippocampal subfield among four groups (IGE VPA1 VS. IGE VPA1 VS. IGE VPA- VS. HCs). The disease duration may also have an impact on hippocampal volume in patients with IGE. To address this, we divided the patients who were currently using VPA into three subgroups based on disease duration: IGE-VPA1 with a disease duration of ≤2 years, IGE-VPA2 with a disease duration of 2–10 years, and IGE-VPA3 with a disease duration of >10 years. Additionally, we have further analyzed the impact of the number of ASMs used in IGE patients (categorized as group IGE1 ASM=0; IGE2, ASM=1; IGE3, ASM≥2) on hippocampal volume.

CAT12 (Computational Anatomy Toolbox 12, CAT12, http://www.neuro.uni-jena.de/cat/), an extension of SPM12 (Statistical Parametric Mapping 12, http://www.fil.ion.ucl.ac.uk/spm/software/spm12/) was used to provide computational anatomy. All the T1-weighted MR images were processed and examined using the CAT12 for segmentation and volume calculations running in MATLAB 2022a (The MathWorks, Inc., Mass., USA) and obtain GM, WM and CSF volumes, which together make up the TIV (Ashburner and Friston, 2005).

### 2.4 Subgroup analyses

To further investigate the volume alterations of hippocampus and its subfields in subsyndromes of IGE, subgroup analyses were performed in patients with JME and GTCA, respectively. While the other two groups (JAE and CAE) were combined as one group, absence epilepsy (AE), to conduct another subgroup analysis.

### 2.5 Statistical analysis

Demographic and clinical data were analyzed using IBM SPSS (version 26). The scanning age, total intracranial volume (TIV) and the hippocampal asymmetry index were compared utilizing independent sample t-tests, while gender distribution was assessed using Chi-square tests. Multivariate Analysis of Covariance (MANCOVA) was employed to investigate group and subgroup differences in total hippocampal and subfield volumes, using age, gender and TIV as covariates, and false discovery rate (FDR) for multiple comparisons correction, which can control the expected proportion of false discoveries while maintaining statistical power, particularly when multiple comparisons are involved. The adjusted alpha level for FDR was computed using the Benjamini-Hochberg procedure, which ranks the p-values and determines the threshold for significance based on the total number of comparisons being made. Partial Eta Squared (η2) was calculated to estimate effect sizes. Spearman correlation tests were conducted to explore the relationships between hippocampal and subfields volumes and clinical characteristics, e.g. age of onset, disease duration, anxiety and depression scores, and frequency of GTCS with FDR-corrected. The association between the dosage or the duration of VPA taken or frequency of GTCS and hippocampal volume in IGE patients currently taking VPA was further investigated using spearman correlation tests. The level of statistical significance was set at *p*<0.05.

## 3 Results

### 3.1 Cohort characteristics

Demographic and clinical characteristics were summarized in Table 1. A total of 211 patients with IGE (mean age 21.0±5.5 years), including JME (n=143), GTCA (n=52), JAE (n=13) and CAE (n=3), as well as 97 HCs (21.5±5.4 years), were enrolled in this study. No significant differences between patients with IGE and HCs were found in age (*p*=0.476), gender distribution (p=0.498), TIV (*p*=0.375) and the hippocampal asymmetry index (*p*=0.673). Ninety-nine patients completed the anxiety and depression Scale assessment (Table S1).

**Table 1:** Demographic data and clinical characteristics of IGE and HCs.

	IGE (n = 211)	HCs (n = 97)	*P* value
Age, mean (SD), years	21.0(5.5)	21.0(5.5)	0.476
Age of onset, mean (SD), years	14.2(3.4)	−	−
Gender, n(female%)	100(47.4)	47(48.5)	0.498
Disease duration, mean (SD), years	6.84(5.48)	−	−
TIV (cm^3^)	1478.01(145.52)	1493.78(142.58)	0.375
Hippocampal Asymmetry Index	0.03(0.05)	0.04(0.04)	0.673
Frequency of GTCS, n			
None	21	−	−
Only once	28	−	−
Less than once a year	75	−	−
Half to once a year	33	−	−
One month to half a year	38	−	−
At least monthly	16	−	−

Abbreviations: IGE, idiopathic generalized epilepsy; HCs: healthy controls; SD, standard deviation; TIV, total intracranial volume; GTCS, generalized tonic-clonic seizure.

### 3.2 Volumetric analysis of IGE and HCs

**Fig. 2:**
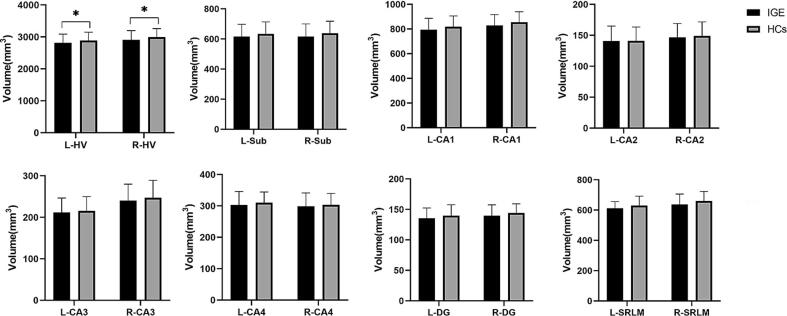
Bar chart of volumes of hippocampal subfields and total hippocampal volume (mm^3^) in patients with in IGE compared with HCs, adjusted for age, sex, and TIV. *Indicates significance after FDR correction. Compared with HCs, the bilateral hippocampal volumes were reduced in IGE (left, F = 4.211, *p* = 0.041; right, F = 5.692, *p* = 0.035). Abbreviations: IGE, idiopathic generalized epilepsy; HCs: healthy controls; HV, hippocampal volume; Sub, subiculum; CA, Cornu Ammonis; DG, dentate gyrus; SRLM, stratum radiatum, Lacunosum, and moleculare.

Hippocampal subfields and total hippocampal volumes in IGE and HCs were summarized in Table 2 and Fig. 2. Compared with HCs, the bilateral hippocampal volumes were reduced in IGE (left, F=4.211, *p*=0.041; right, F=5.692, *p*=0.035). When comparing subfields and total volumes between the left and right hippocampus in patients with IGE, the total volume of the left hippocampus (F=17.437, *p*<0.05), as well as the volumes of the left CA1 (F=18.713, *p*<0.05), CA2(F=7.628, *p*=0.008), CA3(F=79.059, *p*<0.05), DG (F=8.103, *p*=0.008), and SRLM (F=21.118, *p*<0.05), were smaller than those of the right hippocampus in patients (Table S2, Fig. S1). The comparison of subfields and total volumes between the left and right hippocampus in HCs was summarized in Table S3, Fig. S1.

**Table 2:** Hippocampal subfields and total hippocampal volumes (mm^3^) in IGE and HCs. (*p < 0.05, FDR-corrected).

	IGE(n = 211)	HCs(n = 97)	F	Partial etasquared	*P* value (FDR-corrected)
Left hippocampus, Mean (SD)					
Total volume	2815.75(274.05)	2891.38(256.67)	4.211	0.014	0.041*
Sub	616.91(80.96)	634.21(79.74)	1.973	0.006	0.282
CA1	794.91(91.95)	819.38(87.13)	3.663	0.012	0.158
CA2	140.89(24.23)	141.21(22.41)	0.017	0.000	0.896
CA3	211.71(35.00)	215.75(34.23)	0.360	0.001	0.640
CA4	302.91(43.29)	310.25(34.26)	1.659	0.005	0.309
DG	135.49(16.85)	139.93(14.91)	4.097	0.013	0.158
SRLM	612.94(43.29)	630.65(61.45)	4.040	0.013	0.158
Right hippocampus, Mean (SD)					
Total volume	2909.38(287.37)	2996.99(264.05)	5.692	0.018	0.035*
Sub	616.03(84.39)	637.57(80.57)	3.288	0.011	0.165
CA1	829.63(87.27)	855.17(85.55)	4.556	0.015	0.158
CA2	146.76(22.43)	149.09(22.74)	0.215	0.001	0.693
CA3	240.47(39.54)	247.09(42.08)	0.906	0.003	0.479
CA4	298.92(42.63)	303.89(36.19)	0.594	0.002	0.562
DG	139.85(17.76)	144.02(15.16)	2.990	0.010	0.170
SRLM	637.73(68.46)	660.16(63.21)	6.879	0.022	0.128

Abbreviation: IGE, idiopathic generalized epilepsy; HCs: healthy controls; FDR, false discovery rate; SD, standard deviation; Sub, subiculum; CA, Cornu Ammonis; DG, dentate gyrus; SRLM, stratum radiatum, Lacunosum, and moleculare.

### 3.3 Volumetric analysis of effects of VPA

**Fig. 3:**
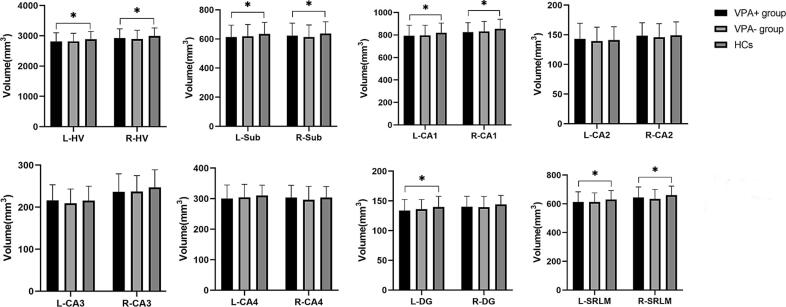
Bar chart of volumes of hippocampal subfields and total hippocampal volume (mm^3^) in VPA + group and VPA- group in IGE compared with HCs, adjusted for age, sex, and TIV. *Indicates significance after FDR correction. Significant reductions were observed in the bilateral hippocampal volumes (left, F = 3.785, *p* = 0.014; right, F = 5.692, *p* = 0.017), as well as in the bilateral subiculum, CA1, SRLM, and left DG in the VPA + group compared with HCs. Abbreviations: VPA, valproate; HCs: healthy controls; HV, hippocampal volume; Sub, subiculum; CA, Cornu Ammonis; DG, dentate gyrus; SRLM, stratum radiatum, Lacunosum, and moleculare.

Demographic data and clinical characteristics of the VPA+ group, the VPA- group in IGE and HCs were summarized in Table S4. Hippocampal subfields and total hippocampal volumes in IGE currently taking VPA (VPA+, n=71, 19 females, mean age=22.2±6.0) and those not currently taking VPA (VPA-, n=140, 92 females, mean age=20.4±5.1) compared with HCs, were summarized in Table 3 and Fig. 3. Significant reductions were observed in the bilateral hippocampal volumes (left, F=3.785, *p*=0.014; right, F=5.692, *p*=0.017), as well as in the bilateral subiculum, CA1, SRLM, and left DG in the VPA+ group compared with HCs. No significant difference in the bilateral hippocampi and subfields was found between VPA+ group and past VPA users (n=111, 75 females, mean age=20.00±5.1 years) or between past VPA users and never VPA users (n=29, 17 females, mean age=22.2±4.9 years). Compared with “never VPA users”, VPA+ group showed significant volume reduction in the right hippocampus (F=2.379, *p*=0.034) and its subfields, including CA1 (F=2.720, *p*=0.034), DG (F=2.877, *p*=0.034), SRLM (F=2.403, *p*=0.034), summarized in Table S5. The median dosage of VPA taken was 500mg (62.5∼1250mg) in the IGE patients currently taking VPA, then patients were divided into two groups: IGE VPA1 dosage ≤500mg/d (n=44) and IGE VPA2 dosage >500mg/d (n=27) summarized in Table S6. The volumes of bilateral hippocampi, CA1 and SRLM, left subiculum and DG were reduced in IGE VPA1 compared with HCs. While no significant hippocampal volume alteration was detected between IGE VPA2, IGE VPA- and HCs. Hippocampal volumes and the volumes of subregions among three groups with different lengths of disease duration were summarized in Table S7. IGE-VPA1 exhibited a reduction in the volume of the left CA4 compared to HCs. Conversely, IGE-VPA2 showed no significant abnormalities, while IGE-VPA3 showed reduced overall left hippocampal volume and the volumes of certain subregions. The results of the impact of the number of ASMs used in IGE patients on hippocampal volume were summarized in Table S8. There was only a significant difference in hippocampal and subregion volumes between IGE3 and HCs. The proportion of patients using VPA in the IGE2 and IGE3 groups was 30.17% (35/116) and 62.07% (36/58), respectively.

**Table 3:** Hippocampal subfields and total hippocampal volumes (mm^3^) in VPA + group and VPA- group in IGE and HCs. (*p < 0.05, FDR-corrected).

	VPA + group (n = 71)	VPA- group (n = 140)	HCs (n = 97)	F	Partial etasquared	*P1, p2, p3, p* value (FDR-corrected)
Left hippocampus, Mean (SD)						
Total volume	2812.93(287.91)	2817.18(268.80)	2891.38(256.67)	3.785	0.024	0.297,0.014*,0.512,0.224
Sub	613.81(81.29)	618.48(81.33)	634.21(79.74)	3.622	0.023	0.176,0.014*,0.690,0.182
CA1	792.84(94.22)	795.96(91.44)	819.38(87.13)	2.904	0.019	0.436,0.014*,0.512,0.233
CA2	143.32(26.16)	139.65(23.28)	141.21(22.41)	0.229	0.002	0.392,0.744,0.926,0.580
CA3	216.49(36.88)	209.28(34.01)	215.75(34.23)	0.319	0.002	0.578,0.744,0.562,0.580
CA4	300.14(44.72)	304.31(42.80)	310.25(34.26)	1.504	0.010	0.424,0.121,0.690,0.330
DG	133.96(18.38)	136.26(16.09)	139.93(14.91)	3.330	0.022	0.297,0.014*,0.512,0.224
SRLM	612.36(71.04)	613.24(62.79)	630.65(61.45)	3.517	0.023	0.297,0.014*,0.512,0.224
Right hippocampus, Mean (SD)						
Total volume	2930.88(300.11)	2898.48(282.22)	2996.99(264.05)	3.531	0.023	0.648,0.017*,0.266,0.619
Sub	622.62(86.62)	612.68(83.66)	637.57(80.57)	2.108	0.014	0.648,0.049*,0.331,0.619
CA1	824.47(84.85)	832.24(88.97)	855.17(85.55)	4.456	0.029	0.628,0.009*,0.331,0.310
CA2	148.39(21.84)	145.94(22.83)	149.09(22.74)	0.134	0.001	0.994,0.578,0.696,0.816
CA3	236.92(42.47)	237.19(37.84)	247.09(42.08)	0.544	0.004	0.752,0.578,0.331,0.795
CA4	303.98(39.95)	296.35(43.99)	303.89(36.19)	0.373	0.002	0.994,0.878,0.467,0.795
DG	140.30(17.52)	139.62(18.00)	144.02(15.16)	1.871	0.012	0.648,0.091,0.331,0.619
SRLM	644.20(73.28)	634.45(66.20)	660.16(63.21)	3.812	0.025	0.648,0.017*,0.231,0.619

Abbreviation: VPA+, patients currently taking VPA; VPA-, patients not currently taking VPA; IGE, idiopathic generalized epilepsy; HCs: healthy controls; FDR, false discovery rate; SD, standard deviation; Sub, subiculum; CA, Cornu Ammonis; DG, dentate gyrus; SRLM, stratum radiatum, Lacunosum, and moleculare. *p*1: VPA + VS. VPA-; *p*2: VPA + VS. HCs; *p*3, VPA- VS. HCs; *p*, significance among three groups.

### 3.4 Subgroup analyses

Hippocampal subfields and total hippocampal volumes in GTCA (n=52), JME (n=143) and AE (n=16) compared with HCs were summarized in Table S9, Table S10 and Table S11, respectively. Significant volume reductions were detected in the right hippocampus (F=5.795, *p*=0.04) and its subfields, including the Sub (F=6.284, *p*=0.04), DG (F=5.525, *p=*0.04) and SRLM (F=7.070, *p*<0.05) in the GTCA group.

### 3.5 Correlation analysis

A negative correlation was observed between the left CA2 and the age of onset (r=-0.215, p=0.016, FDR-corrected) in patients with IGE, as summarized in Table S12. However, disease duration, frequency of GTCS, anxiety and depression scores (Table S13) did not demonstrate any significant correlation with the volumes of the bilateral hippocampi and corresponding subfields in IGE, as evidenced by our findings in this study. Moreover, in the VPA+ group, no significant correlation was found between the volumes of the bilateral hippocampi and the dosage (625.18±246.65mg, 62.5∼1250mg), or the duration (5.01±5.39years, 0.08∼23years) of VPA taken or frequency of GTCS (Table S14).

## 4 Discussion

Our study emphasized the structural alterations of the hippocampus and its subfields in patients with IGE compared with HCs. The key finding identified that the volume reductions of hippocampi and subfields were associated with current VPA use. Additionally, we observed hippocampal volumetric asymmetry within patients with IGE. Subgroup analyses showed significant volume reductions in right hippocampus and its subfields in the GTCA group compared with HCs. Moreover, a negative correlation was found between the left CA2 and the age of onset. These findings, together with previous studies (Crespo Pimentel et al., 2024, Shin et al., 2024); suggest that VPA may have broader neuroanatomical effects in IGE, potentially explaining the heterogeneity observed in neuroimaging studies of IGE.

Previous studies (Caciagli et al., 2019, Zhou et al., 2015) mainly focused on overall hippocampal volume changes instead of the subfields of the hippocampus in patients with IGE. Our study applied HippUnfold (DeKraker et al., 2022); a newly published automated segmentation program, to explore the clinical relevance of volume changes in the hippocampal subfields in patients with IGE. HippUnfold uses U-net learning to take into account the variable folding patterns of the hippocampus, including the head and the tail of the hippocampus, and segments the subfields through the long axis of the hippocampus, which respects the different internal hippocampal folding configurations seen between individuals and is more flexible to T1WI or T2 WI data, sub-millimetric isotropic or thick-slice anisotropic data, compared with some notable software widely used in the previous studies, including Freesurfer (Sämann et al., Jan 2022) and Automatic Segmentation of Hippocampus subfield (ASHS) (Xie et al., 2019). Previous neuroimaging studies have reported volume reductions of the left (Caciagli et al., 2019, Tae et al., 2006) or right (Lin et al., Oct 2013) or even bilateral (Kim et al., Aug 2015) hippocampi in JME compared with HCs. Marked hippocampal atrophy on the left (Bochyńska et al., Dec 2022) or bilateral (Zhou et al., Feb 2015) were also detected in GTCA. In our study, using HippUnfold, we identified bilateral hippocampal atrophy in IGE, with subgroup analysis revealing significant volume reductions in right hippocampus and its subfields, e.g. Sub, DG and SRLM in GTCA instead of those in JME or AE compared to HCs. This suggested heterogeneity in hippocampal volume reductions among IGE subtypes, since previous neuroimaging studies included different subtypes of IGE. The heterogeneity in IGE imaging findings may also arise from several factors, including differences in sample characteristics (such as sample size, age, gender and comorbidities), imaging protocols, and methodologies used in volume analysis. In light of these observations, it may be beneficial to include more imaging studies exploring hippocampal volume changes in IGE patients in future *meta*-analyses. This could provide a more comprehensive understanding of the structural alterations associated with IGE and further elucidate the factors contributing to heterogeneity in previous studies.

In addition, the abnormalities in both the hippocampal subfields and the whole hippocampus were associated with current VPA use in our study. Previous studies mainly focused on the effects of VPA during its administration and its impact on brain structure. Prior morphometric MRI studies indicated that VPA use was linked to bilateral precentral gyri (Crespo Pimentel et al., 2024) and parieto-occipitals (Pardoe et al., 2013, Tondelli et al., 2020) cortical thinning, as well as GM volume reductions in the frontal cortex and cerebellum (Shin et al., Jan 2024) in heterogeneous epilepsy syndrome. The potential mechanism of cortical changes observed in patients with epilepsy undergoing VPA treatment remains unknown. Studies have indicated that prenatal exposure to VPA can disrupt neuronal proliferation and differentiation, leading to neocortical dysgenesis in both mice (Fujimura et al., 2016) and human cortical organoids (Cui et al., 2020). Additionally, VPA exposure may reduce cortical and brainstem volumes or abnormal gyrification of brain in animal studies (Frisch et al., 2009, Sawada et al., 2021). Remarkably, individuals in the VPA+ group in the current study exhibited notable volume reductions not only in the bilateral hippocampi, but also in hippocampal subfields compared with HCs. We also found VPA+ group showed significant right hippocampal atrophy and its subfields, including CA1, DG and SRLM compared with never VPA users, while no significant difference was detected between VPA+ group and past VPA users or between past VPA users and never VPA users, which suggested that VPA effects were somewhat reversible. The potential mechanisms for this reversibility may be the neuroadaptation or the restoration of neurogenesis following the cessation of VPA treatment. Further longitudinal comparison study with pre- and post-medication brain structural changes is warranted to validate this finding. Previous study reported delayed pruning in JME in particular, which may affect cortical grey matter volume (Lin et al., Nov 2014). Interestingly, there was no correlation between the disease duration and the volume of the bilateral hippocampi and their subfields in IGE, even though we investigated the impact of disease duration on hippocampal volume. It may indicate that the hippocampal volume is not directly influenced by the duration of the disease during its progression and other factors might play a more significant role in affecting hippocampal volume, such as the pathophysiological changes of the disease and neuroinflammatory responses. We also investigated the potential impact of ASMs on the hippocampal volume. As the number of ASM increased, volume of the hippocampus and its subregions gradually decreased. Although there was a significant difference in hippocampal and subregion volumes between “IGE3” group (those taking ≥2 ASMs) and HCs. We further analyzed the proportion of patients using VPA in the “IGE2” and “IGE3” groups. We found that 30.17% of patients in “IGE2” group using VPA, while 62.07% in “IGE3” group, indicating a higher proportion of VPA users in the “IGE3” group. We speculate that VPA may play a certain role in altering the volumes of the hippocampus and its subregions. Meanwhile, dosage or duration of VPA taken and GTCS frequency of GTCS were not correlated with hippocampal volume in VPA+ group. The current VPA+ group had relatively low dosage and short duration of VPA use, as well as relatively low frequency of GTCS among our included patients, with 76.2% (54/71) had a GTCS frequency of less than once every six months. We then detected that the volumes of bilateral hippocampi, CA1 and SRLM, left subiculum and dentate gyrus were reduced in patients with dosage of VPA taken≤500mg compared with HCs, suggesting that a lower dosage of VPA taken contributed to a more significant volume reduction in hippocampal volume than a higher dosage of VPA taken. This seemed to further strengthen our results that there was no dose correlation. Additionally, factors such as age, gender, and genetic predispositions may contribute to the inconsistence. Despite the use of different tools or methodologies, we all observed a reduction in hippocampal volume in IGE patients using VPA, which suggested the crucial consideration of medication use in future imaging studies of IGE. Moreover, a longitudinal design of future study is warranted to explore the relationship between VPA use and the observed hippocampal volume changes.

Notably, the volume of the left CA2 was observed to be negatively correlated with age of onset in IGE. Most previous studies showed no correlation between the hippocampus volume and clinical characteristics, such as disease duration and age of onset in patients with JME (Caciagli et al., 2019, Tae et al., 2006, Kim et al., 2015). Another study including 40 patients with JME found that the disease duration was correlated with left hippocampus volume while age of onset correlated with right hippocampus volume (Saini et al., Apr 2013). According to previous studies, CA2 is associated with psychiatric disorders such as manic depression, schizophrenia, autism spectrum disorder and temporal lobe epilepsy (Modi et al., 2019, Benes et al., 1998, Whitebirch et al., 2022, Benes et al., 2001). The volume reduction of left CA2 was more common in older patients indicating that CA2 is distinct from other subdivisions of the hippocampus and showing more atrophy as patients getting older. Understanding the role of CA2 subfield in relation to psychiatric conditions and epilepsy could be crucial for further exploring the significance of these structural findings in patients with IGE.

Additionally, several previous studies have explored the relationship between the hippocampus or its subregions and anxiety or depression (Chen et al., Feb 2019, Monereo-Sánchez et al., 2024, Roddy et al., 2019). In our study, no correlation was found between anxiety or depression scores and volume of both the hippocampi and subfields. Further investigations with a larger sample size compared with HCs may also be needed to explore potential factors contributing to the absence of correlation between anxiety, depression, and hippocampal volumes in individuals with IGE. Psychiatric disorders are common in patients with IGE and may significantly influence both brain structure and treatment response. It would be interesting to include patients with other psychiatric comorbidities as well and study the effects associated the medical treatment and brain functions. What’s more, additional neuropsychological tests such as working memory tests could be conducted to correlate with structural alterations of the hippocampus in IGE.

The main strength of the current research is that this study represents the first attempt to segment the hippocampus in IGE using HippUnfold, a newly published and validated automated segmentation program. Besides, we compared the volume of the whole and subfields of the hippocampus and their association with clinical characteristics within and between two groups with a relatively large sample size. This study also has several limitations. Firstly, the lack of longitudinal follow-up in this study impedes the ability to discern the causal relationship between antiepileptic drug use and hippocampal volume changes over time. Longitudinal studies are essential to validate and extend the findings from this cross-sectional analysis and to delineate the long-term effects of VPA on hippocampal morphology in IGE patients. Secondly, the absence of assessment pertaining to the potential influence of concomitant medications alongside VPA on hippocampal volumes in IGE patients poses a significant limitation. Understanding the combined effects of multiple antiepileptic drugs may provide a more comprehensive insight into the impact on hippocampal structure in this patient population. Furthermore, the lack of comparative data between IGE patients and HCs on anxiety and depression measures impedes a comprehensive understanding of the psychological well-being and mental health status in both groups. Finally, the impact of psychosocial variables and lifestyle factors, such as stress levels, sleep quality, and physical activity, on hippocampal volume in IGE patients may not have been fully accounted for in the current study. Consideration these factors in future research could yield a more comprehensive understanding of the multifaceted contributors to hippocampal volume alterations in epilepsy.

## 5 Conclusions

The current study revealed bilateral hippocampal atrophy, segmented by HippUnfold, in a relatively large number of patients with IGE compared to HCs. Our findings demonstrated a notable correlation between current VPA use and alterations in hippocampal volume and subfield volumes in patients with IGE. Future longitudinal studies are essential to validate these findings and to provide a more comprehensive understanding of the distinct effects of VPA on hippocampal morphology in individuals with IGE. By highlighting the correlation between VPA use and hippocampal volume alterations, these findings suggest that VPA may have more extensive neuroanatomical effects in IGE, potentially accounting for the heterogeneity observed in neuroimaging studies of IGE.

## CRediT authorship contribution statement

**Xiang Huang:** Writing – original draft, Visualization, Methodology, Investigation, Formal analysis, Data curation, Conceptualization. **Yingying Zhang:** Writing – review & editing, Visualization, Supervision, Funding acquisition, Formal analysis, Data curation, Conceptualization. **Qiuxing Lin:** Software, Resources, Methodology, Conceptualization. **Kailing Huang:** Software, Resources, Methodology, Conceptualization. **Yuming Li:** Data curation, Conceptualization. **Peiwen Liu:** Data curation, Conceptualization. **Danyang Cao:** Data curation, Conceptualization. **Wenhao Li:** Data curation, Conceptualization. **Wei Li:** Data curation, Conceptualization. **Xiuli Li:** Validation, Data curation, Conceptualization. **Qiyong Gong:** Validation, Data curation, Conceptualization. **Dong Zhou:** Visualization, Validation, Supervision. **Dongmei An:** Writing – review & editing, Visualization, Validation, Supervision, Software, Project administration, Methodology, Investigation, Funding acquisition, Formal analysis, Data curation, Conceptualization.

## Funding

This study was supported by the National Natural Science Foundation of China (NSFC Grants No.82101521, 82171443, 82471482, 81771402, U21A20393), Key project of Science and technology department of Sichuan Province (Grants No. 2024YFFK0316), The 1.3.5 Talent Excellence Development Project − West China Hospital of Sichuan University (Grants No. ZYGD23032), The 1.3.5 project for disciplines of excellence and Brain Science project of West China Hospital, Sichuan University (Grants No. ZYJC21001) and National Key R&D Program Project (Grants No. 2021YFC2401204).

## Declaration of Competing Interest

The authors declare that they have no known competing financial interests or personal relationships that could have appeared to influence the work reported in this paper.

## Data Availability

Data will be made available on request.
