# Supplementary material for: Association of valproate use and hippocampal atrophy in idiopathic generalized epilepsy
Source: Neuroimage Clin. 2025 Jan 27;45:103744. doi: 10.1016/j.nicl.2025.103744 (PMC11847523; doi:10.1016/j.nicl.2025.103744)
Supplement: Supplementary Data 1 [file mmc1.docx]

Association of valproate use and hippocampal atrophy in Idiopathic Generalized Epilepsy

Table S1 Anxiety and Depression scores in patients with IGE.

|  | IGE (n=99) |
| --- | --- |
| Age, mean (SD), years | 21.31(5.49) |
| Gender, n(female%) | 53(53.5%) |
| Disease duration, mean (SD), years | 7.21(5.71) |
| TIV (cm^3^) | 1474.19(158.99) |
| HAMA score, mean (SD) | 6.64(7.21) |
| HAMD score, mean (SD) | 10.05(11.11) |

Abbreviations: IGE, idiopathic generalized epilepsy; SD, standard deviation; TIV, total intracranial volume; HAMA, Hamilton Anxiety Rating Scale; HAMD, Hamilton Depression Rating Scale.

Table S2 Comparison of left and right hippocampal subfields and total hippocampal volume (mm^3^) in IGE. (**p* < 0.05, FDR-corrected)

|  | Left,  Mean (SD) | Right,  Mean (SD) | F | Partial eta squared | *P* value (FDR-corrected) |
| --- | --- | --- | --- | --- | --- |
| Total volume | 2815.75(274.05) | 2909.38(287.37) | 17.437 | 0.040 | <0.05* (7.2365e-05) |
| Sub | 616.91(80.96) | 616.03(84.39) | 0.016 | 0.000 | 0.899 |
| CA1 | 794.91(91.95) | 829.63(87.27) | 18.713 | 0.043 | <0.05* (5.0748e-05) |
| CA2 | 140.89(24.23) | 146.76(22.43) | 7.628 | 0.018 | 0.008* |
| CA3 | 211.71(35.00) | 240.47(39.54) | 79.059 | 0.159 | <0.05*(0) |
| CA4 | 302.91(43.29) | 298.92(42.63) | 1.007 | 0.002 | 0.369 |
| DG | 135.49(16.85) | 139.85(17.76) | 8.103 | 0.019 | 0.008* |
| SRLM | 612.94(43.29) | 637.73(68.46) | 21.118 | 0.048 | <0.05*(2.2940e-05) |

Abbreviation: IGE, idiopathic generalized epilepsy; SD, standard deviation; Sub, subiculum; CA, Cornu Ammonis; DG, dentate gyrus; SRLM, stratum radiatum, Lacunosum, and moleculare.

Table S3 Comparison of left and right hippocampal subfields and total hippocampal volume (mm^3^) in HCs. (**p* < 0.05, FDR-corrected)

|  | Left,  Mean (SD) | Right,  Mean (SD) | F | Partial eta squared | *P* value  (FDR-corrected) |
| --- | --- | --- | --- | --- | --- |
| Total volume | 2891.38(256.67) | 2996.99(264.05) | 11.127 | 0.056 | 0.003* |
| Sub | 634.21(79.74) | 637.57(80.57) | 0.114 | 0.001 | 0.736 |
| CA1 | 819.38(87.13) | 855.17(85.55) | 10.334 | 0.052 | 0.003* |
| CA2 | 141.21(22.41) | 149.09(22.74) | 6.855 | 0.035 | 0.015* |
| CA3 | 215.75(34.23) | 247.09(42.08) | 36.811 | 0.163 | <0.05* |
| CA4 | 310.25(34.26) | 303.89(36.19) | 1.569 | 0.008 | 0.242 |
| DG | 139.93(14.91) | 144.02(15.16) | 3.775 | 0.020 | 0.071 |
| SRLM | 630.65(61.45) | 660.16(63.21) | 14.350 | 0.071 | <0.05* |

Abbreviation: HCs: healthy controls; SD, standard deviation; Sub, subiculum; CA, Cornu Ammonis; DG, dentate gyrus; SRLM, stratum radiatum, Lacunosum, and moleculare.

Table S4 Demographic data and clinical characteristics of VPA+ group and VPA- group in IGE and HCs. (**p*<0.05)

|  | IGE VPA (+)  (n=71) | IGE VPA (-)  (n=140) | HC(n=97) | *P* value |
| --- | --- | --- | --- | --- |
| Age, mean (SD), years | 22.2(6.0) | 20.4(5.1) | 21.5(5.4) | 0.061 |
| Gender, n(female%) | 19(26.8%) | 92(65.7%) | 47(48.5%) | <0.05 |
| Disease duration, mean (SD), years | 8.69(6.12) | 5.91(4.89) | / | 0.041* |
| TIV (cm3) | 1509.72(127.04) | 1461.93(147.43) | 1493.78(142.58) | 0.051 |
| Age of onset, mean (SD), years | 13.6(2.7) | 14.5(3.6) | / | 0.079 |
| Duration of VPA taken, mean (SD), years | 5.01(5.39) | / | / | / |
| Dosage of VPA taken, mean (SD), mg | 625.18(246.65) | / | / | / |

Abbreviations: VPA, valproate; IGE, idiopathic generalized epilepsy; HCs: healthy controls; SD, standard deviation; TIV, total intracranial volume.

Table S5 Hippocampal subfields and total hippocampal volumes (mm3) in VPA+, past VPA users, never VPA users and HCs. (**p* < 0.05, FDR-corrected)

|  | VPA+ group  (n=71) | Past VPA users (n=111)  Mean (SD) | Never VPA users (n=29)  Mean (SD) | HCs  (n=97) | F | Partial eta squared | *P1, p2, p3, p4, p5, p6* value (FDR-corrected) |
| --- | --- | --- | --- | --- | --- | --- | --- |
| Left hippocampus, Mean (SD) | | | | | | | |
| Total volume | 2812.93(287.91) | 2795.45(283.66) | 2900.35(183.16) | 2891.38(256.67) | 3.690 | 0.035 | 0.319,0.542,0.014*,0.082,0.255,1.3158e-05* |
| Sub | 613.81(81.29) | 613.25(81.98) | 638.47(76.89) | 634.21(79.74) | 3.822 | 0.06 | 0.443,0.115,0.014*,0.200,0.407,3.8150e-08* |
| CA1 | 792.84(94.22) | 791.51(96.29) | 812.98(68.57) | 819.38(87.13) | 1.145 | 0.011 | 0.319,0.937,0.014*,0.200,0.274,0.071 |
| CA2 | 143.32(26.16) | 138.62(23.91) | 143.58(20.58) | 141.21(22.41) | 1.735 | 0.017 | 0.443,0.937,0.744,0.200,0.620,0.144 |
| CA3 | 216.49(36.88) | 206.97(34.43) | 218.12(31.38) | 215.75(34.23) | 1.650 | 0.016 | 0.515,0.115,0.744,0.182,0.255,0.016* |
| CA4 | 300.14(44.72) | 302.38(42.70) | 311.71(43.12) | 310.25(34.26) | 1.399 | 0.013 | 0.376,0.513,0.121.0.200,0.407,0.067 |
| DG | 133.96(18.38) | 135.57(16.63) | 138.92(13.78) | 139.93(14.91) | 1.820 | 0.017 | 0.374,0.748,0.014*,0.200,0.255,0.040* |
| SRLM | 612.36(71.04) | 607.14(65.78) | 636.57(43.18) | 630.65(61.45) | 4.648 | 0.043 | 0.376,0.542,0.014*,0.056,0.255,4.3179e-05* |
| Right hippocampus, Mean (SD) | | | | | | | |
| Total volume | 2930.88(300.11) | 2879.80(296.05) | 2969.96(210.66) | 2996.99(264.05) | 2.379 | 0.023 | 0.945,0.034*,0.017*,0.161,0.080,2.2787e-06* |
| Sub | 622.62(86.62) | 607.84(87.63) | 631.23(64.30) | 637.57(80.57) | 1.437 | 0.014 | 0.945,0.068,0.049*,0.168,0.124,2.2787e-06* |
| CA1 | 824.47(84.85) | 827.08(93.12) | 851.99(68.73) | 855.17(85.55) | 2.720 | 0.026 | 0.897,0.034*,0.009*,0.168,0.170,1.2749e-04* |
| CA2 | 148.39(21.84) | 146.42(24.01) | 144.08(17.88) | 149.09(22.74) | 0.074 | 0.001 | 0.945,0.999,0.578,0.827,0.829,1.5747e-04* |
| CA3 | 236.92(42.47) | 234.62(39.04) | 247.05(31.52) | 247.09(42.08) | 1.669 | 0.016 | 0.897,0.224,0.578,0.168,0.163, 0.003* |
| CA4 | 303.98(39.95) | 295.83(46.34) | 298.36(34.12) | 303.89(36.19) | 0.045 | 0.000 | 0.945,0.999,0.878,0.877,0.506,0.932 |
| DG | 140.30(17.52) | 138.22(18.72) | 144.99(13.95) | 144.02(15.16) | 2.877 | 0.027 | 0.945,0.034*,0.091,0.161,0.124,0.017* |
| SRLM | 644.20(73.28) | 629.80(68.92) | 652.26(51.80) | 660.16(63.21) | 2.403 | 0.023 | 0.945,0.034*,0.017*,0.161,0.057,3.0428e-05* |

Abbreviation: VPA+, patients currently taking VPA; HCs: healthy controls; FDR, false discovery rate; SD, standard deviation; Sub, subiculum; CA, Cornu Ammonis; DG, dentate gyrus; SRLM, stratum radiatum, Lacunosum, and moleculare; *p*1: VPA+ VS. past VPA users; *p*2: VPA+ VS. never VPA users; *p*3,VPA+ VS. HCs; *p*4, past VPA users VS. never VPA users; *p*5, past VPA users VS. HCs; *p*6, never VPA users VS. HCs.

Table S6 Hippocampal subfields and total hippocampal volumes (mm3) in different dosage of VPA in IGE and HCs. (**p* < 0.05, FDR-corrected)

|  | IGE VPA1  (≤500mg)  (n=44) | IGE VPA2  (>500mg)  (n=27) | IGE VPA-  (n=140) | HCs  (n=97) | F | Partial eta squared | *P1, p2, p3, p4, p5* value (FDR-corrected) |
| --- | --- | --- | --- | --- | --- | --- | --- |
| Left hippocampus, Mean (SD) | | | | | | | |
| Total volume | 2757.89(270.80) | 2902.61(297.35) | 2817.18(268.80) | 2891.38(256.67) | 3.167 | 0.031 | 0.823,0.151,0.005*,0.938,0.425 |
| Sub | 597.09(71.92) | 641.07(89.40) | 618.48(81.33) | 634.21(79.74) | 3.070 | 0.030 | 0.979,0.059,0.005*,0.921,0.566 |
| CA1 | 775.93(96.17) | 820.40(85.64) | 795.96(91.44) | 819.38(87.13) | 2.610 | 0.025 | 0.823,0.254,0.006*,0.921,0.425 |
| CA2 | 143.29(28.47) | 143.37(22.41) | 139.65(23.28) | 141.21(22.41) | 0.237 | 0.002 | 0.979,0.254,0.686,0.921,0.720 |
| CA3 | 213.68(37.88) | 221.07(35.41) | 209.28(34.01) | 215.75(34.23) | 0.221 | 0.002 | 0.979,0.584,0.799,0.921,0.844 |
| CA4 | 298.54(45.25) | 302.75(44.56) | 304.31(42.80) | 310.25(34.26) | 1.002 | 0.010 | 0.979,0.458,0.135,0.921,0.425 |
| DG | 132.32(18.13) | 136.64(18.82) | 136.26(16.09) | 139.93(14.91) | 2.306 | 0.022 | 0.979,0.254,0.015*,0.921,0.425 |
| SRLM | 597.05(68.02) | 637.14(69.95) | 613.24(62.79) | 630.65(61.45) | 3.454 | 0.033 | 0.979,0.123,0.005*,0.921,0.569 |
| Right hippocampus, Mean (SD) | | | | | | | |
| Total volume | 2879.41(296.79) | 3014.77(291.58) | 2898.48(282.22) | 2996.99(264.05) | 2.688 | 0.026 | 0.978,0.566,0.016*,0.979,0.443 |
| Sub | 615.53(78.85) | 634.1(98.47) | 612.68(83.66) | 637.57(80.57) | 1.410 | 0.014 | 0.978,0.782,0.103,0.979,0.443 |
| CA1 | 806.48(83.65) | 853.79(79.83) | 832.24(88.97) | 855.17(85.55) | 3.786 | 0.036 | 0.978,0.252,0.005*,0.979,0.443 |
| CA2 | 144.99(22.88) | 153.93 (19.16) | 145.94(22.83) | 149.09(22.74) | 0.362 | 0.004 | 0.978,0.782,0.378,0.979,0.885 |
| CA3 | 240.23(43.84) | 257.82(38.40) | 237.19(37.84) | 247.09(42.08) | 0.725 | 0.007 | 0.978,0.981,0.378,0.903,0.885 |
| CA4 | 301.89(40.60) | 307.38(39.39) | 296.35(43.99) | 303.89(36.19) | 0.248 | 0.002 | 0.978,0.981,0.721,0.979,0.885 |
| DG | 138.08(17.12) | 143.91 (17.88) | 139.62(18.00) | 144.02(15.16) | 1.399 | 0.014 | 0.978,0.566,0.077,0.979,0.885 |
| SRLM | 632.20(73.71) | 663.76(69.51) | 634.45(66.20) | 660.16(63.21) | 2.832 | 0.027 | 0.978,0.566,0.016*,0.979,0.443 |

Abbreviation: IGE, idiopathic generalized epilepsy; VPA-, patients not currently taking VPA; HCs: healthy controls; FDR, false discovery rate; SD, standard deviation; Sub, subiculum; CA, Cornu Ammonis; DG, dentate gyrus; SRLM, stratum radiatum, Lacunosum, and moleculare; *p*1: IGE VPA1 VS. IGE VPA2; *p*2: IGE VPA1 VS. IGE VPA-; *p*3, IGE VPA1 VS. HCs; *p*4, IGE VPA2 VS. IGE VPA-; *p*5, IGE VPA2 VS. HCs.

Table S7 Hippocampal subfields and total hippocampal volumes (mm3) in three different lengths of disease duration groups in IGE-VPA and HCs. (**p* < 0.05, FDR-corrected)

|  | IGE-VPA1  (≤2y)  (n=10) | IGE-VPA2  (2-10y)  (n=37) | IGE-VPA3 (>10y)  (n=24) | HCs  (n=97) | F | Partial eta squared | *P1, p2, p3, p4, p5, p6* value (FDR-corrected) |
| --- | --- | --- | --- | --- | --- | --- | --- |
| Left hippocampus, Mean (SD) | | | | | | | |
| Total volume | 2795.67(411.78) | 2838.51(293.02) | 2780.67(221.97) | 2891.38(256.67) | 4.132 | 0.071 | 0.314,0.851,0.133,0.761,0.372,0.035* |
| Sub | 625.77(71.19) | 619.79(88.98) | 599.63(73.53) | 634.21(79.74) | 3.177 | 0.056 | 0.398,0.851,0.186,0.821,0.372,0.071 |
| CA1 | 792.41(143.98) | 790.82(90.00) | 796.14(78.81) | 819.38(87.13) | 2.527 | 0.045 | 0.759,0.851,0.186,0.761,0.372,0.044* |
| CA2 | 142.81(39.90) | 144.59(24.11) | 141.58(23.31) | 141.21(22.41) | 0.350 | 0.006 | 0.645,0.851,0.726,0.761,0.587,0.741 |
| CA3 | 218.70(38.93) | 216.26(33.75) | 215.91(41.98) | 215.75(34.23) | 0.218 | 0.004 | 0.517,0.851,0.585,0.821,0.827,0.925 |
| CA4 | 272.68(51.61) | 310.95(43.48) | 294.92(39.25) | 310.25(34.26) | 4.855 | 0.083 | 0.03*,0.259,0.018,0.821,0.827,0.086 |
| DG | 131.69(21.59) | 135.89(18.54) | 131.94(17.20) | 139.93(14.91) | 3.236 | 0.057 | 0.398,0.851,0.186,0.821,0.372,0.035* |
| SRLM | 611.61(101.67) | 620.21(72.28) | 600.56(53.77) | 630.65(61.45) | 3.756 | 0.065 | 0.314,0.851,0.174,0.761,0.372,0.035* |
| Right hippocampus, Mean (SD) | | | | | | | |
| Total volume | 2905.74(433.24) | 2945.72(290.17) | 2918.49(260.94) | 2996.99(264.05) | 3.946 | 0.068 | 0.074,0.657,0.150.0.849,0.333,0.100 |
| Sub | 624.67(88.62) | 619.80(97.74) | 626.11(69.16) | 637.57(80.57) | 2.031 | 0.036 | 0.350,0.657,0.221,0.849,0.333,0.409 |
| CA1 | 813.43(133.51) | 825.39(71.87) | 827.66(82.40) | 855.17(85.55) | 4.281 | 0.074 | 0.162,0.657,0.150,0.849,0.333,0.088 |
| CA2 | 147.07(28.14) | 150.51(19.73) | 145.66(22.74) | 149.09(22.74) | 1.214 | 0.022 | 0.103,0.657,0.337,0.849,0.773,0.422 |
| CA3 | 244.91(45.64) | 249.54(42.83) | 243.72(42.20) | 247.09(42.08) | 0.808 | 0.015 | 0.103,0.657,0.337,0.849,0.930,0.699 |
| CA4 | 288.29(33.48) | 310.64(37.24) | 300.25(45.35) | 303.89(36.19) | 1.379 | 0.025 | 0.009*,0.112,0.337,0.849,0.533,0.787 |
| DG | 139.00(22.69) | 141.69(16.72) | 138.70(16.99) | 144.02(15.16) | 1.784 | 0.032 | 0.182,0.455,0.337,0.849,0.698,0.243 |
| SRLM | 648.37(114.62) | 648.15(69.43) | 636.38(59.68) | 660.16(63.21) | 3.295 | 0.058 | 0.103,0.657,0.221,0.849,0.333,0.088 |

Abbreviation: IGE, idiopathic generalized epilepsy; VPA, valproate; HCs: healthy controls; FDR, false discovery rate; SD, standard deviation; Sub, subiculum; CA, Cornu Ammonis; DG, dentate gyrus; SRLM, stratum radiatum, Lacunosum, and moleculare; *p*1: IGE-VPA1 VS. IGE-VPA2; *p*2: IGE-VPA1 VS. IGE-VPA3; *p*3, IGE-VPA1 VS. HCs; *p*4, IGE-VPA2 VS. IGE-VPA3; *p*5, IGE-VPA2 VS. HCs; *p*6, IGE-VPA3 VS. HCs.

Table S8 Hippocampal subfields and total hippocampal volumes (mm3) in three groups using different number of ASMs in IGE and HCs. (**p* < 0.05, FDR-corrected)

|  | IGE1 (ASM=0)  (n=37) | IGE2 (ASM=1)  (n=116) | IGE3  (ASM≥2)  (n=58) | HCs  (n=97) | F | Partial eta squared | *P1, p2, p3, p4, p5, p6* value (FDR-corrected) |
| --- | --- | --- | --- | --- | --- | --- | --- |
| Left hippocampus, Mean (SD) | | | | | | | |
| Total volume | 2862.57(298.52) | 2847.65(266.48) | 2722.07(256.98) | 2891.38(256.67) | 3.229 | 0.031 | 0.979,0.185,0.826,0.083,0.432,0.003* |
| Sub | 620.50(92.97) | 625.42(80.20) | 597.58(72.75) | 634.21(79.74) | 1.250 | 0.012 | 0.979,0.398,0.826,0.220,0.616,0.051 |
| CA1 | 808.61(93.41) | 802.89(91.45) | 770.21(89.68) | 819.38(87.13) | 2.322 | 0.023 | 0.979,0.252,0.826,0.220,0.432,0.010* |
| CA2 | 143.72(24.48) | 142.61(23.84) | 135.63(24.67) | 141.21(22.41) | 0.662 | 0.007 | 0.979,0.447,0.826,0.220,0.698,0.357 |
| CA3 | 213.26(36.96) | 216.45(35.33) | 201.23(31.53) | 215.75(34.23) | 1.555 | 0.015 | 0.979,0.398,0.826,0.083,0.769,0.054 |
| CA4 | 311.54(47.98) | 302.61(42.88) | 298.01(41.24) | 310.25(34.26) | 0.992 | 0.010 | 0.979,0.398,0.826,0.984,0.432,0.168 |
| DG | 140.90(17.49) | 136.86(15.98) | 129.29(16.75) | 139.93(14.91) | 4.512 | 0.043 | 0.979,0.080,0.826,0.0831,0.432,0.003* |
| SRLM | 624.04(66.46) | 620.81(65.17) | 590.12(60.98) | 630.65(61.45) | 3.386 | 0.033 | 0.979,0.185,0.826,0.083,0.432,0.003* |
| Right hippocampus, Mean (SD) | | | | | | | |
| Total volume | 2967.33(320.89) | 2938.92(278.79) | 2813.34(165.53) | 2996.99(264.05) | 3.732 | 0.036 | 0.742,0.134,0.883.0.109,0.240,0.002* |
| Sub | 610.41(96.37) | 629.96(82.66) | 591.75(75.50) | 637.57(80.57) | 2.918 | 0.028 | 0.414,0.641,0.441,0.109,0.659,0.009* |
| CA1 | 858.22(95.80) | 832.88(87.71) | 804.87(75.50) | 855.17(85.55) | 3.646 | 0.035 | 0.414,0.060,0.883,0.370,0.240,0.006* |
| CA2 | 151.90(24.66) | 146.95(21.06) | 143.12(23.49) | 149.09(22.74) | 0.627 | 0.006 | 0.485,0.355,0.883,0.864,0.643,0.486 |
| CA3 | 246.08(46.39) | 244.56(37.99) | 228.71(36.34) | 247.09(42.08) | 1.413 | 0.014 | 0.742,0.245,0.883,0.144,0.754,0.046 |
| CA4 | 303.85(42.95) | 298.80(43.68) | 296.01(41.09) | 303.89(36.19) | 0.323 | 0.003 | 0.742,0.665,0.883,0.998,0.643,0.416 |
| DG | 145.67(19.33) | 141.04(16.12) | 133.74(18.57) | 144.02(15.16) | 4.034 | 0.039 | 0.414,0.060,0.883,0.109,0.366,0.006* |
| SRLM | 651.20(75.69) | 644.73(66.14) | 615.14(64.66) | 660.16(63.21) | 4.089 | 0.039 | 0.742,0.142,0.883,0.109,0.240,0.002* |

Abbreviation: IGE, idiopathic generalized epilepsy; ASM, antiseizure medication; HCs: healthy controls; FDR, false discovery rate; SD, standard deviation; Sub, subiculum; CA, Cornu Ammonis; DG, dentate gyrus; SRLM, stratum radiatum, Lacunosum, and moleculare; *p*1: IGE1 VS. IGE2; *p*2: IGE VS. IGE3; *p*3, IGE1 VS. HCs; *p*4, IGE2 VS. IGE3; *p*5, IGE2 VS. HCs; *p*6, IGE3 VS. HCs.

Table S9 Hippocampal subfields and total hippocampal volumes (mm^3^) in GTCA and HCs. (**p* < 0.05, FDR-corrected)

|  | GTCA(n=52) | HCs(n=97) | F | Partial eta squared | *P* value (FDR-corrected) |
| --- | --- | --- | --- | --- | --- |
| Left hippocampus, Mean (SD) | | | | | |
| Total volume | 2801.80(275.32) | 2891.38(256.67) | 2.770 | 0.019 | 0.358 |
| Sub | 611.33(80.70) | 634.21(79.74) | 2.134 | 0.015 | 0.358 |
| CA1 | 792.90(87.41) | 819.38(87.13) | 1.386 | 0.010 | 0.358 |
| CA2 | 140.25(25.09) | 141.21(22.41) | 0.011 | 0.000 | 0.916 |
| CA3 | 210.30(25.09) | 215.75(34.23) | 0.585 | 0.004 | 0.510 |
| CA4 | 301.28(45.96) | 310.25(34.26) | 1.453 | 0.010 | 0.358 |
| DG | 133.58(16.81) | 139.93(14.91) | 4.448 | 0.030 | 0.294 |
| SRLM | 612.15(67.19) | 630.65(61.45) | 2.133 | 0.015 | 0.358 |
| Right hippocampus, Mean (SD) | | | | | |
| Total volume | 2876.54(293.58) | 2996.99(264.05) | 5.795 | 0.039 | 0.04* |
| Sub | 602.23(79.32) | 637.57(80.57) | 6.284 | 0.042 | 0.04* |
| CA1 | 822.71(83.27) | 855.17(85.55) | 3.059 | 0.021 | 0.132 |
| CA2 | 147.27(24.18) | 149.09(22.74) | 0.023 | 0.000 | 0.879 |
| CA3 | 239.81(40.86) | 247.09(42.08) | 0.392 | 0.003 | 0.608 |
| CA4 | 298.35(50.02) | 303.89(36.19) | 0.448 | 0.003 | 0.608 |
| DG | 136.87(18.87) | 144.02(15.16) | 5.525 | 0.037 | 0.04* |
| SRLM | 629.29(70.24) | 660.16(63.21) | 7.070 | 0.047 | 0.04* |

Abbreviation: GTCA, generalized tonic–clonic seizures alone; HCs: healthy controls; FDR, false discovery rate; SD, standard deviation; Sub, subiculum; CA, Cornu Ammonis; DG, dentate gyrus; SRLM, stratum radiatum, Lacunosum, and moleculare.

Table S10 Hippocampal subfields and total hippocampal volumes (mm^3^) in JME and HCs. (**p* < 0.05, FDR-corrected)

|  | JME (n=143) | HCs (n=97) | F | Partial eta squared | *P* value (FDR-corrected) |
| --- | --- | --- | --- | --- | --- |
| Left hippocampus, Mean (SD) | | | | | |
| Total volume | 2822.30(274.05) | 2891.38(256.67) | 2.736 | 0.012 | 0.265 |
| Sub | 619.39(82.81) | 634.21(79.74) | 0.974 | 0.004 | 0.433 |
| CA1 | 796.68(91.31) | 819.38(87.13) | 2.760 | 0.012 | 0.265 |
| CA2 | 141.36(24.69) | 141.21(22.41) | 0.113 | 0.000 | 0.737 |
| CA3 | 212.01(35.00) | 215.75(34.23) | 0.188 | 0.001 | 0.737 |
| CA4 | 303.78(42.96) | 310.25(34.26) | 1.014 | 0.004 | 0.433 |
| DG | 136.25(17.49) | 139.93(14.91) | 2.075 | 0.009 | 0.302 |
| SRLM | 612.84(65.47) | 630.65(61.45) | 3.360 | 0.014 | 0.265 |
| Right hippocampus, Mean (SD) | | | | | |
| Total volume | 2926.22(286.41) | 2996.99(264.05) | 2.656 | 0.011 | 0.279 |
| Sub | 622.71(88.46) | 637.57(80.57) | 0.829 | 0.004 | 0.582 |
| CA1 | 833.39(86.31) | 855.17(85.55) | 2.696 | 0.011 | 0.279 |
| CA2 | 146.71(21.56) | 149.09(22.74) | 0.170 | 0.001 | 0.680 |
| CA3 | 241.53(39.38) | 247.09(42.08) | 0.443 | 0.002 | 0.599 |
| CA4 | 299.35(41.40) | 303.89(36.19) | 0.407 | 0.002 | 0.599 |
| DG | 141.04(17.96) | 144.02(15.16) | 1.075 | 0.005 | 0.582 |
| SRLM | 641.48(67.77) | 660.16(63.21) | 3.538 | 0.015 | 0.279 |

Abbreviation: JME, juvenile myoclonic epilepsy; HCs: healthy controls; FDR, false discovery rate; SD, standard deviation; Sub, subiculum; CA, Cornu Ammonis; DG, dentate gyrus; SRLM, stratum radiatum, Lacunosum, and moleculare.

Table S11 Hippocampal subfields and total hippocampal volumes (mm3) in AE and HCs. (**p* < 0.05, FDR-corrected

|  | AE(n=16) | HCs(n=97) | F | Partial eta squared | *P* value (FDR-corrected) |
| --- | --- | --- | --- | --- | --- |
| Left hippocampus, Mean (SD) | | | | | |
| Total volume | 2788.52(341.20) | 2891.38(256.67) | 3.334 | 0.030 | 0.319 |
| Sub | 619.35(83.27) | 634.21(79.74) | 0.809 | 0.007 | 0.443 |
| CA1 | 776.49(127.06) | 819.38(87.13) | 4.275 | 0.038 | 0.319 |
| CA2 | 136.03(28.76) | 141.21(22.41) | 0.904 | 0.008 | 0.443 |
| CA3 | 210.53(33.12) | 215.75(34.23) | 0.461 | 0.004 | 0.515 |
| CA4 | 299.01(43.10) | 310.25(34.26) | 1.433 | 0.013 | 0.376 |
| DG | 134.28(13.69) | 139.93(14.91) | 2.206 | 0.020 | 0.374 |
| SRLM | 612.83(78.29) | 630.65(61.45) | 1.713 | 0.016 | 0.376 |
| Right hippocampus, Mean (SD) | | | | | |
| Total volume | 2878.05(360.08) | 2996.99(264.05) | 4.381 | 0.039 | 0.215 |
| Sub | 609.24(83.91) | 637.57(80.57) | 2.813 | 0.025 | 0.215 |
| CA1 | 820.37(118.18) | 855.17(85.55) | 2.995 | 0.027 | 0.215 |
| CA2 | 145.05(28.07) | 149.09(22.74) | 0.566 | 0.005 | 0.447 |
| CA3 | 234.57(44.77) | 247.09(42.08) | 1.689 | 0.015 | 0.336 |
| CA4 | 296.14(34.21) | 303.89(36.19) | 0.724 | 0.007 | 0.447 |
| DG | 140.02(15.74) | 144.02(15.16) | 1.057 | 0.010 | 0.413 |
| SRLM | 632.65(88.92) | 660.16(63.21) | 3.912 | 0.035 | 0.215 |

Abbreviation: AE, absence epilepsy; HCs: healthy controls; FDR, false discovery rate; SD, standard deviation; Sub, subiculum; CA, Cornu Ammonis; DG, dentate gyrus; SRLM, stratum radiatum, Lacunosum, and moleculare.

Table S12 Correlation of hippocampal subfields and total hippocampal volume with disease duration, age of onset and frequency of GTCS in IGE. (**p* < 0.05, FDR-corrected)

| Disease duration | L-HV | L-Sub | L-CA1 | L-CA2 | L-CA3 | L-CA4 | L-DG | L-SRLM |
| --- | --- | --- | --- | --- | --- | --- | --- | --- |
| r | 0.030 | 0.019 | 0.063 | 0.007 | 0.059 | -0.038 | -0.001 | 0.004 |
| *p* value | 0.666 | 0.784 | 0.364 | 0.916 | 0.398 | 0.587 | 0.989 | 0.950 |
|  | R-HV | R-Sub | R-CA1 | R-CA2 | R-CA3 | R-CA4 | R-DG | R-SRLM |
| r | 0.048 | 0.046 | 0.072 | -0.008 | 0.057 | 0.012 | 0.004 | 0.015 |
| *p* value | 0.484 | 0.504 | 0.296 | 0.905 | 0.407 | 0.867 | 0.955 | 0.826 |
| Age of onset | L-HV | L-Sub | L-CA1 | L-CA2 | L-CA3 | L-CA4 | L-DG | L-SRLM |
| r | -0.091 | 0.022 | -0.078 | -0.215 | -0.068 | -0.107 | -0.103 | -0.086 |
| *p* value | 0.188 | 0.749 | 0.260 | 0.016* | 0.327 | 0.122 | 0.137 | 0.214 |
|  | R-HV | R-Sub | R-CA1 | R-CA2 | R-CA3 | R-CA4 | R-DG | R-SRLM |
| r | -0.104 | -0.047 | -0.109 | -0.095 | -0.023 | -0.144 | -0.063 | -0.091 |
| *p* value | 0.131 | 0.501 | 0.115 | 0.169 | 0.743 | 0.037 | 0.360 | 0.188 |
| Frequency of GTCS | L-HV | L-Sub | L-CA1 | L-CA2 | L-CA3 | L-CA4 | L-DG | L-SRLM |
| r | -0.063 | 0.003 | -0.123 | 0.082 | 0.004 | 0.007 | -0.045 | -0.057 |
| *p* value | 0.366 | 0.968 | 0.074 | 0.237 | 0.954 | 0.923 | 0.520 | 0.413 |
|  | R-HV | R-Sub | R-CA1 | R-CA2 | R-CA3 | R-CA4 | R-DG | R-SRLM |
| r | -0.083 | 0.001 | -0.174 | -0.110 | -0.028 | -0.007 | 0.001 | -0.071 |
| *p* value | 0.230 | 0.987 | 0.011 | 0.110 | 0.684 | 0.921 | 0.987 | 0.306 |

Abbreviation: GTCA, generalized tonic–clonic seizures alone; IGE, idiopathic generalized epilepsy; L, left; R, right; Sub, subiculum; CA, Cornu Ammonis; DG, dentate gyrus; SRLM, stratum radiatum, Lacunosum, and moleculare.

Table S13 Correlation of hippocampal subfields and total hippocampal volume with anxiety and depression scores in IGE. (*p < 0.05, FDR-corrected)

| HAMA | LHV | LSub | LCA1 | LCA2 | LCA3 | LCA4 | LDG | LSRLM |
| --- | --- | --- | --- | --- | --- | --- | --- | --- |
| r | -0.130 | -0.060 | -0.160 | -0.120 | -0.160 | 0.070 | -0.090 | -0.150 |
| *p* value | 0.200 | 0.540 | 0.120 | 0.240 | 0.120 | 0.490 | 0.370 | 0.130 |
|  | RHV | RSub | RCA1 | RCA2 | RCA3 | RCA4 | RDG | RSRLM |
| r | -0.120 | -0.120 | -0.100 | -0.050 | -0.140 | -0.040 | -0.110 | -0.110 |
| *p* value | 0.220 | 0.240 | 0.320 | 0.640 | 0.160 | 0.710 | 0.270 | 0.270 |
| HAMD | LHV | LSub | LCA1 | LCA2 | LCA3 | LCA4 | LDG | LSRLM |
| r | -0.150 | -0.080 | -0.170 | -0.080 | -0.170 | 0.040 | -0.110 | -0.190 |
| *p* value | 0.130 | 0.440 | 0.090 | 0.460 | 0.100 | 0.690 | 0.270 | 0.060 |
|  | RHV | RSub | RCA1 | RCA2 | RCA3 | RCA4 | RDG | RSRLM |
| r | -0.170 | -0.180 | -0.120 | -0.080 | -0.140 | -0.110 | -0.170 | -0.170 |
| *p* value | 0.090 | 0.080 | 0.240 | 0.460 | 0.170 | 0.300 | 0.100 | 0.090 |

Abbreviation: IGE, idiopathic generalized epilepsy; L, left; R, right; Sub, subiculum; CA, Cornu Ammonis; DG, dentate gyrus; SRLM, stratum radiatum, Lacunosum, and moleculare; HAMA, Hamilton Anxiety Rating Scale; HAMD, Hamilton Depression Rating Scale.

Table S14 Correlation of hippocampal subfields and total hippocampal volume with the dosage or the duration of VPA taken and frequency of GTCS in IGE currently taking VPA. (**p* < 0.05, FDR-corrected)

| Dosage of VPA taken | L-HV | L-Sub | L-CA1 | L-CA2 | L-CA3 | L-CA4 | L-DG | L-SRLM |
| --- | --- | --- | --- | --- | --- | --- | --- | --- |
| r | 0.252 | 0.270 | 0.200 | 0.063 | 0.128 | 0.070 | 0.103 | 0.284 |
| *p* value | 0.034 | 0.023 | 0.094 | 0.600 | 0.287 | 0.560 | 0.392 | 0.016 |
|  | R-HV | R-Sub | R-CA1 | R-CA2 | R-CA3 | R-CA4 | R-DG | R-SRLM |
| r | 0.268 | 0.138 | 0.294 | 0.224 | 0.269 | 0.096 | 0.205 | 0.270 |
| *p* value | 0.024 | 0.253 | 0.013 | 0.061 | 0.023 | 0.426 | 0.087 | 0.023 |
| Duration of VPA taken | L-HV | L-Sub | L-CA1 | L-CA2 | L-CA3 | L-CA4 | L-DG | L-SRLM |
| r | -0.098 | -0.103 | -0.036 | -0.121 | -0.072 | -0.013 | -0.040 | -0.133 |
| *p* value | 0.415 | 0.395 | 0.769 | 0.313 | 0.549 | 0.915 | 0.741 | 0.268 |
|  | R-HV | R-Sub | R-CA1 | R-CA2 | R-CA3 | R-CA4 | R-DG | R-SRLM |
| r | -0.108 | -0.026 | -0.056 | -0.152 | -0.128 | -0.082 | -0.098 | -0.160 |
| *p* value | 0.369 | 0.831 | 0.641 | 0.207 | 0.288 | 0.497 | 0.414 | 0.184 |
| Frequency of GTCS | L-HV | L-Sub | L-CA1 | L-CA2 | L-CA3 | L-CA4 | L-DG | L-SRLM |
| r | -0.063 | 0.003 | -0.123 | 0.082 | 0.004 | 0.007 | -0.045 | -0.057 |
| *p* value | 0.366 | 0.968 | 0.074 | 0.237 | 0.954 | 0.923 | 0.520 | 0.413 |
|  | R-HV | R-Sub | R-CA1 | R-CA2 | R-CA3 | R-CA4 | R-DG | R-SRLM |
| r | -0.083 | 0.001 | -0.174 | -0.110 | -0.028 | -0.007 | 0.001 | -0.071 |
| *p* value | 0.230 | 0.987 | 0.011 | 0.110 | 0.684 | 0.921 | 0.987 | 0.306 |

Abbreviation: VPA, valproate; GTCS, generalized tonic–clonic seizures; IGE, idiopathic generalized epilepsy; L, left; R, right; Sub, subiculum; CA, Cornu Ammonis; DG, dentate gyrus; SRLM, stratum radiatum, Lacunosum, and moleculare.


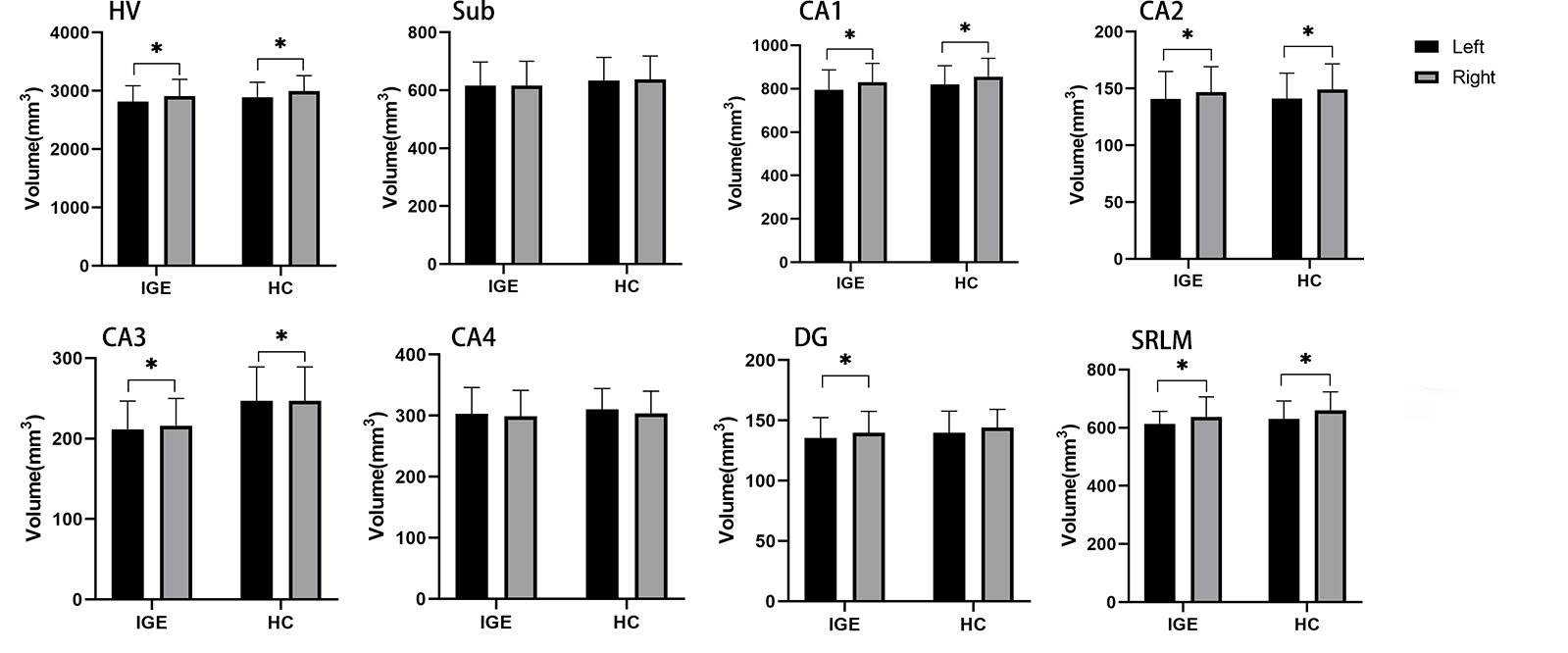


Fig.S1 Bar chart of comparison of left and right hippocampal subfields in patient with IGE and HCs, adjusted for age, sex, and TIV. *Indicates significance after FDR correction. IGE, idiopathic generalized epilepsy; HC: healthy control; HV, hippocampal volume; Sub, subiculum; CA, Cornu Ammonis; DG, dentate gyrus; SRLM, stratum radiatum, Lacunosum, and moleculare.
